# Ultrasound-Guided Clavipectoral Fascial Plane Block for Surgery Involving the Clavicle: A Case Series

**DOI:** 10.7759/cureus.9072

**Published:** 2020-07-08

**Authors:** Promil Kukreja, Camille J Davis, Lisa MacBeth, Joel Feinstein, Hari Kalagara

**Affiliations:** 1 Anesthesiology and Perioperative Medicine, University of Alabama at Birmingham, Birmingham, USA

**Keywords:** sensory innervation, clavipectoral fascial plane block, peri-operative analgesia, regional anesthesia, brachial plexus, cervical plexus

## Abstract

The clavipectoral fascial plane block (CPB) is a novel regional anesthesia technique that has been utilized for clavicular fracture surgery. While the cutaneous innervation of the skin above the clavicle is well-known to be supplied by the supraclavicular nerve of the superficial cervical plexus (SCP), the sensory innervation of the clavicle itself is somewhat controversial. Despite this controversy, it has been hypothesized that the CPB is an effective regional anesthesia technique for peri-operative analgesia since the terminal branches of many of the sensory nerves like suprascapular, subclavian, lateral pectoral, and long thoracic nerves pass through the plane between the clavipectoral fascia and the clavicle itself.

## Introduction

Clavicle fractures account for 2.6% of all fractures and are frequently encountered in both the emergency department and operating room settings [1]. The clavipectoral fascial plane block (CPB) is a novel regional anesthesia technique that has been utilized for clavicular fracture surgeries. Valdés-Vilches originally described the CPB in 2017 as an injection of 10-15 cc of local anesthetic under ultrasound guidance in between the clavipectoral fascia and the periosteum on the medial and lateral aspects of the area of clavicular injury [Presentation: Valdés-Vilches LF. Analgesia for Clavicular Surgery/Fractures. Symposia 01: Postoperative Analgesia for Orthopedic Upper and Lower Limb Surgery: Symposium conducted at the 36th Annual European Society of Regional Anaesthesia and Pain Therapy (ESRA) Congress, Lugano, Switzerland; September 13-16] Since this initial report, a handful of additional case reports have been published that support the efficacy of this block for clavicular surgery [2-5].

In comparison to other surgical sites of the upper extremity, the clavicle has a complex and variable innervation that is continuing to be elucidated, and it has led to numerous discussions as to which regional anesthetic technique is best suited for preventing postoperative pain in clavicle repairs. While the cutaneous innervation of the skin above the clavicle is well-known to be supplied by the supraclavicular nerve of the superficial cervical plexus (SCP), the sensory innervation of the clavicle itself is somewhat controversial. While some sources state that the clavicle is similarly supplied by the supraclavicular nerve of the SCP, other sources maintain that sensation is provided by branches of the brachial plexus such as the subclavian, long thoracic, and suprascapular nerves [6]. Due to this controversy, multiple different regional anesthesia techniques have been utilized in clavicular surgery, including most commonly the SCP block, brachial plexus blocks such as the interscalene, or a combination of the two. While multiple case reports support the use of these techniques [7-9], it can be time-consuming to perform two separate ultrasound-guided injections, and brachial plexus blocks have their own known set of adverse events. These include the almost invariable ipsilateral phrenic nerve palsy with interscalene blocks [10], somewhat common Horner's syndrome or vocal cord paralysis, and the more rare, albeit serious, adverse events of vertebral artery injection, total spinal anesthesia, and pneumothorax [11].

The CPB has proved to be an attractive alternative to the above given its singular injection, ease to perform, and advanced safety profile, especially for patients with respiratory disease. While further studies on this regional anesthetic technique’s safety are needed, no adverse events were noted in the above case reports or this case series. Compared to brachial plexus blocks such as the interscalene block, which prevent pain transmission more proximally and hence lie in close proximity to the neurovascular structures of the cervical spine and neck, the CPB likely offers an improved safety advantage due to its more lateral and superficial plane of injection with the clavicle serving as a natural backstop. It is hypothesized that the CPB provides pain relief by blocking many of the above nerves as their terminal branches pass through the plane between the clavipectoral fascia and the clavicle itself. This case series adds to the growing amount of evidence supporting the CPB through its description of three patients, including one adolescent, who received preoperative CPB for clavicular surgery.

## Case presentation

Three patients undergoing surgical procedures involving the clavicle were identified on the day of their surgeries as CPB candidates. Consent was obtained from these patients prior to surgery. The block was performed preoperatively under minimal sedation with required intravenous midazolam and fentanyl boluses with noninvasive blood pressure measurement every three minutes and continuous electrocardiogram, pulse oximetry, and capnography. Follow-up data were obtained from chart review of the electronic medical records. Informed consent was obtained for the submission of a case report.

**Figure 1:**
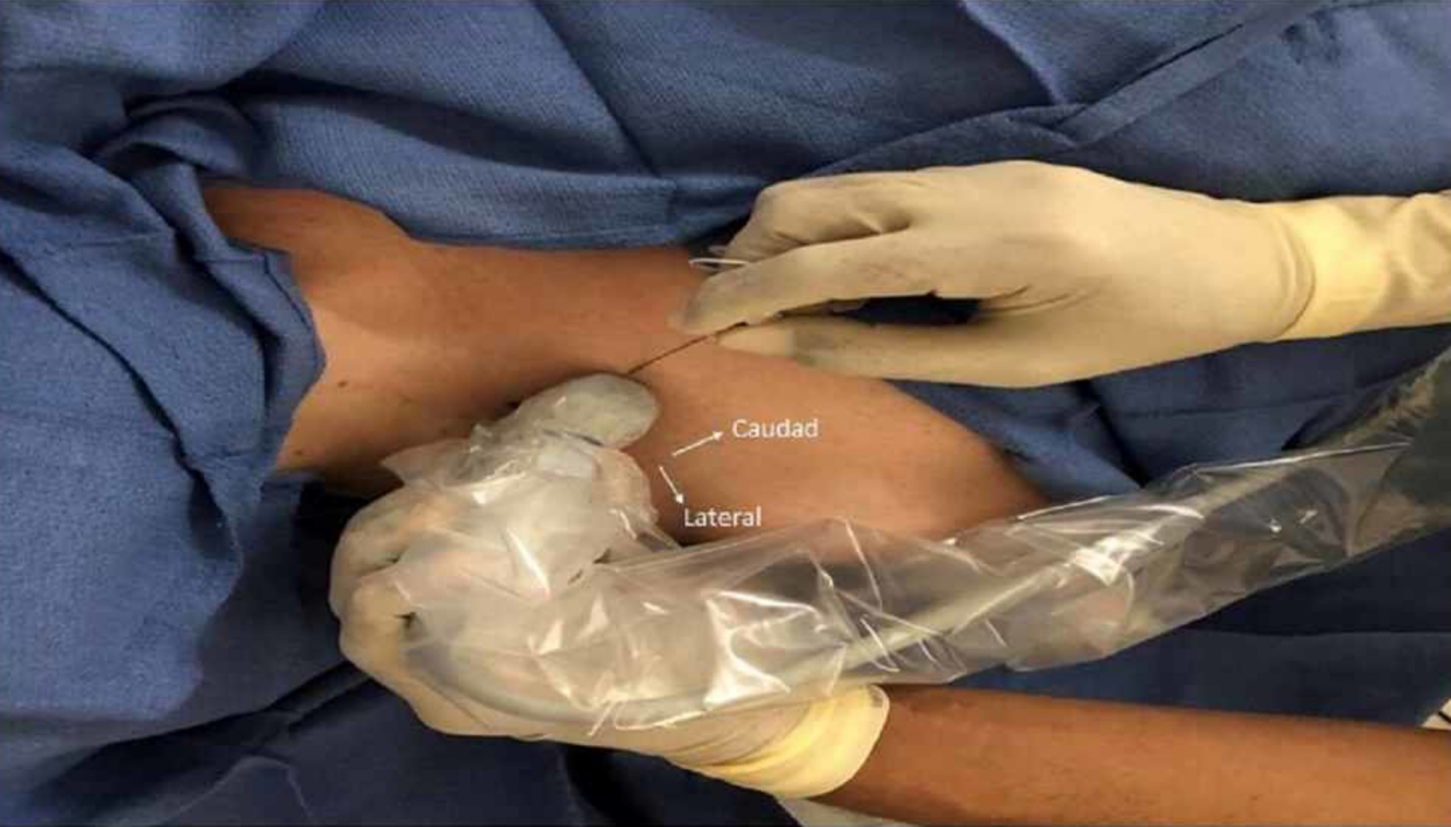
Patient positioning and in-plane needle insertion from caudad to cephalad direction

A CPB was performed using a high-frequency linear probe (Sonosite HFL38x/13-6 MHz; Fujifilm SonoSite, Bothell, WA) placed on the anterior surface of the clavicle. An in-plane technique was used to visualize needle advancing in a caudal to cephalad direction (Figure 1).

**Figure 2:**
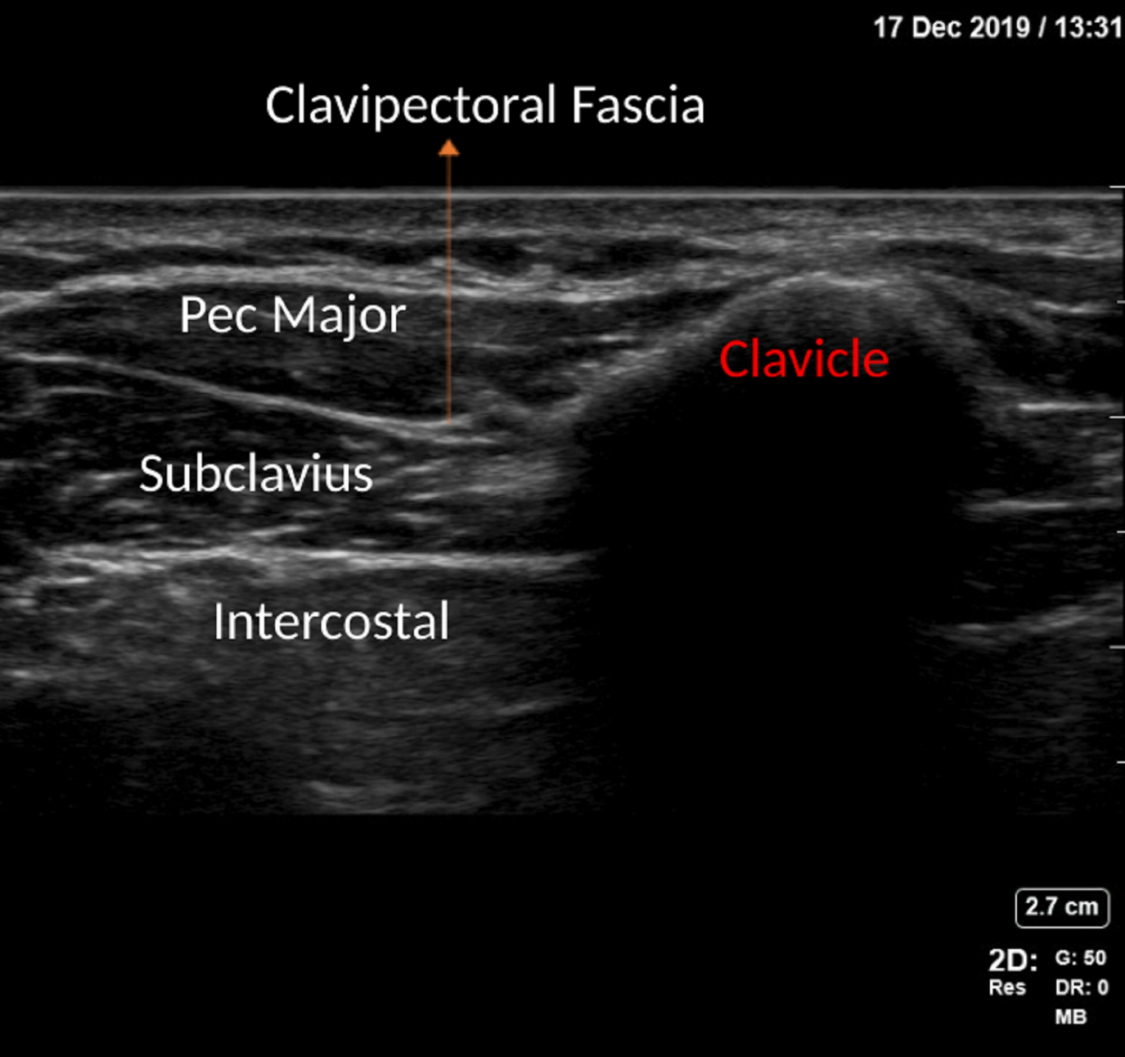
Ultrasound landmarks to identify clavipectoral fascia

**Figure 3:**
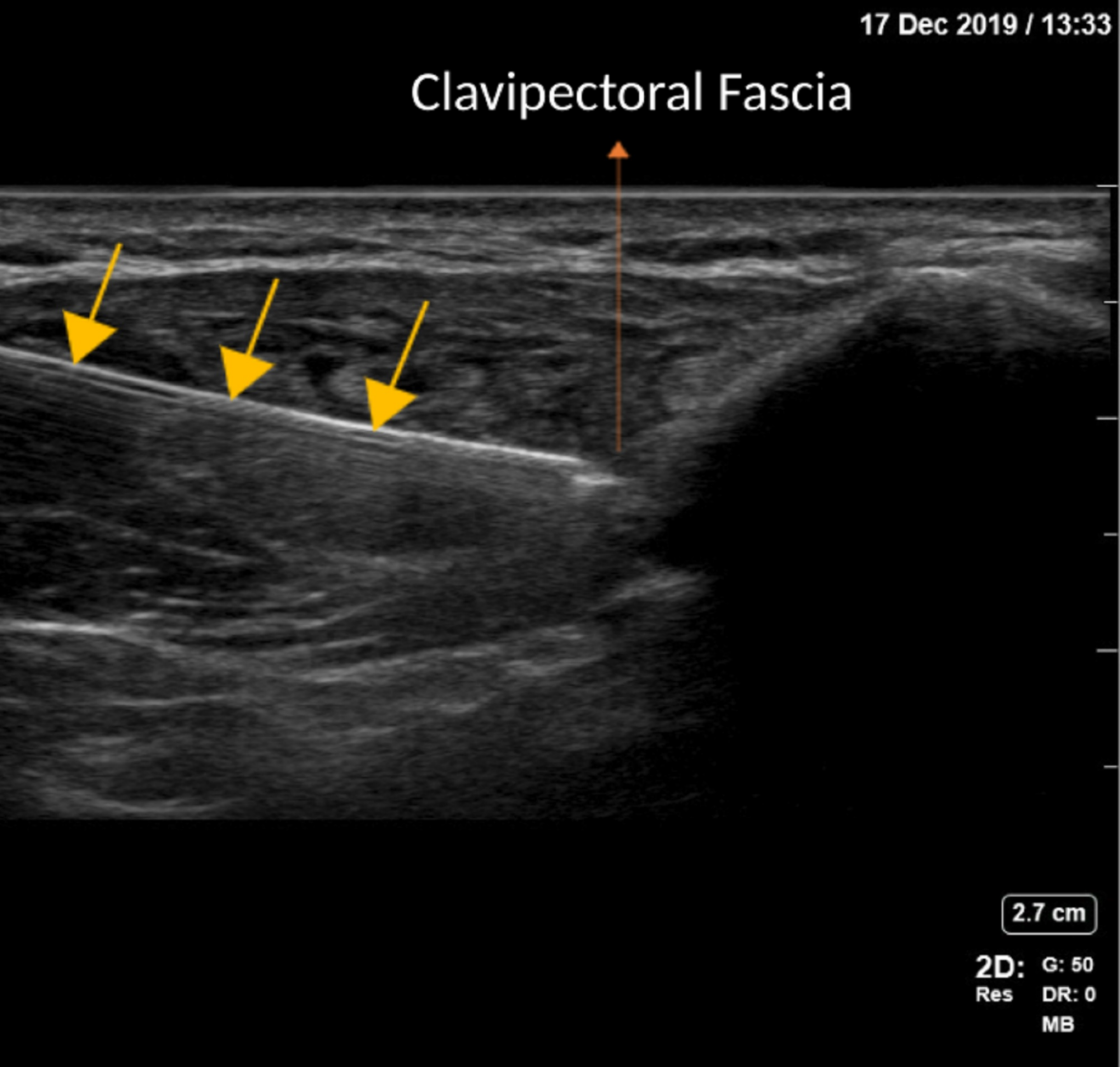
Ultrasound image of in-plane needle technique for CPB CPB: clavipectoral fascial plane block

The ultrasound image of the anatomical landmarks for the CPB is shown in Figure 2, and the ultrasound image of the in-plane needle path for the CPB is shown in Figure 3.

Case studies

Case Study No. 1

A 32-year-old female with a history of hypertension and attention-deficit/hyperactivity disorder presented for right open distal clavicle excision for right acromioclavicular joint arthritis. The CPB was administered using an in-plane technique to visualize an 8-cm, 20-g Tuohy needle advancing in a caudal to the cephalic direction; 15 cc 0.5% ropivacaine was injected in between the clavipectoral fascia and the periosteum of the clavicle. Given the distal nature of the surgery, a right interscalene catheter was also placed with the same ultrasound probe/needle, and 10 cc 0.5% ropivacaine was given as an initial bolus with a continuous infusion of 0.2% ropivacaine solution at 8 cc/hr started postoperatively. No complications were noted during surgery under a general anesthetic, and pain scores remained zero in the post-anesthesia care unit (PACU). No opioids were given in the PACU, and the patient was discharged home after a 90-minute PACU stay. The patient reported 10/10 satisfaction with her regional anesthetic on the routine post-discharge phone follow-up.

Case Study No. 2

A 14-year-old male without other known past medical history underwent open removal of hardware in the right sternoclavicular joint after repair of posterior dislocation with operative reduction and internal fixation of clavicle after encountering a sports-related injury five months ago. The implanted hardware and surgical incision were located over the proximal half of the clavicle. A right CPB was performed in a similar fashion as above with a high-frequency linear ultrasound probe and a 5-cm, 22-g short bevel needle; 15 cc 0.5% ropivacaine with 2 mg of dexamethasone was injected into the appropriate fascial plane. There were no complications during surgery under a general anesthetic. Pain scores remained zero in the PACU, and no additional analgesic medication was administered in the PACU. The patient was discharged home after a routine 120-minute PACU stay. On a routine post-discharge phone follow-up, his guardian reported no complications.

Case Study No. 3

A 19-year-old female with no past medical history underwent left operative reduction and internal fixation of the clavicle after a traumatic mid-shaft clavicular fracture from a motor vehicle collision 17 days prior to surgery. Of note, the patient was not opioid-naïve and had been discharged from the trauma service with a prescription for thirty oxycodone 10 mg tablets 16 days prior to surgery. A CPB was performed in a similar fashion as above preoperatively. A high-frequency linear ultrasound probe and a 5-cm, 22-g short bevel needle were used to inject 10 cc 0.5% ropivacaine both on the medial and lateral side of the mid-shaft fracture. No complications were noted during surgery under a general anesthetic. Pain scores were a 6 on PACU presentation and 5 on PACU discharge. The patient did require intravenous pain medications during a 90-minute PACU stay. The patient did not complain about surgical-site pain but was complaining of severe back pain secondary to recent accident-related injuries. The patient was discharged home from PACU and reported 10/10 satisfaction with her regional anesthetic on a routine post-discharge phone follow-up (Table 1).

**Table 1:** Individual patient-reported pain scores, satisfaction scores, and oral morphine equivalents required in PACU and post-discharge (first 24 hours) *Patient-reported pain scores out of 10 were obtained by PACU nursing staff **Patient satisfaction scores were obtained by daily phone calls after discharge by regional anesthesia residents until sensory/motor blockade resolved. Patients were asked about any complications as well as their subjective satisfaction with their regional anesthetic ***Unable to obtain as the pediatric patient’s guardians participated in phone calls PACU: post-anesthesia care unit

Case study	Patient-reported pain scores*		Oral morphine equivalents required		Patient satisfaction scores**
	On PACU admission	On PACU discharge	During 2-hour PACU stay	During first 24 hours after discharge	
1	0	0	0	7.5	10/10
2	0	0	0	7.5	-***
3	6	5	49.5	30	10/10

## Discussion

The clavicle innervation is complex and controversial due to a lack of validated cadaver studies. The clavicle and anterior superior shoulder area derive its nerve supply from both cervical and brachial plexus. Thus, any single block is usually insufficient to provide effective surgical or peri-operative analgesia. Regional anesthesia for sole clavicle surgeries has not been commonly practiced due to multiple innervations of the clavicle. The combination of ultrasound-guided interscalene and SCP block has been successfully used for open reduction and internal fixation of clavicle fracture [12]. Also, ultrasound-guided SCP and superior trunk interscalene blocks have been used as sole anesthetic techniques for acromioclavicular joint fixation surgery [13].

It has been shown that a large volume of local anesthetic for interscalene brachial plexus block often results in cervical plexus blockade [14]. Low-volume brachial plexus block in addition to low-volume selective supraclavicular nerve block has been reported for clavicle surgeries [15]. Both interscalene and cervical plexus block have the potential to cause phrenic nerve paralysis, which can lead to detrimental effects in some patient populations, such as those with obstructive sleep apnea, obesity, or significant underlying lung disease. Besides diaphragmatic hemiparesis, interscalene block can also cause upper extremity motor block and complications like Horner’s syndrome and adverse events of epidural or vertebral artery injection [11].

All the sensory nerves supplying the clavicle penetrate the clavipectoral fascia to innervate clavicle except for the supraclavicular nerve, which innervates skin above the clavicle. Since the sensory block of skin incision could not be achieved with the CPB, a supraclavicular block (cervical plexus) should be performed [16]. Interestingly, the CPB proved effective in preventing pain despite not performing a separate block of the SCP to provide blockade of the sensory innervation of the skin. Given the superficial nature of the plane of local anesthetic injection during the CPB and its proximity to the skin itself, it is possible that either distal branches of the innervation to the skin also passes between the clavipectoral fascia and the clavicle or diffusion of the local anesthetic-provided cutaneous blockade. The infiltration of local anesthetic at the incision site by surgeons could also potentially block supraclavicular nerve.

These three cases illustrate the efficacy of the CPB as a sole or adjunct peripheral nerve block in clavicle surgery. Two out of the three patients had zero pain after surgery with the third patient having a maximum pain score of 6, demonstrating the efficacy of this block in relieving postoperative pain. All three patients were able to be discharged from the PACU in less than an hour, and two out of two of the patients surveyed reported 10/10 satisfaction with the block. Regional anesthesia results in opioid-sparing with effective analgesia, decreases postoperative nausea and vomiting, and enables early ambulation and discharge [17].

This report discusses the first case in which the CPB has been reported to be utilized safely and efficaciously in an adolescent patient (case no. 2). Since males under the age of 20 experience the most number of sports-related injuries that cause clavicular fractures [1], the CPB is likely to be highly utilized in this population. The CPB use in the pediatric population will clearly provide benefits of ease of performance and avoidance of phrenic nerve and upper extremity weakness. The CPB block can also be used for trauma patients with rib fractures and pneumothorax where general anesthesia may increase the risk of pneumothorax expansion and associated complications [18].

Further studies are needed to clarify the cutaneous distribution of sensory blockade provided by the CPB. Additionally, we propose that the use of an interscalene peripheral nerve block/catheter in addition to the CPB block alone may be helpful in more distal clavicle surgeries involving the acromioclavicular joint. Until the controversy regarding sensory innervation of the clavicle is addressed or randomized controlled trials comparing SCP and brachial plexus blocks in clavicle surgery are performed, a consensus on the regional anesthetic technique to be used for these surgeries may not be reached.

## Conclusions

The CPB provides another effective alternate regional anesthesia technique while avoiding undesirable side effects of more proximal techniques such as motor blockade and phrenic nerve paralysis. Moreover, It does not carry any risk of pneumothorax. The decision to use the CPB alone or in addition to other techniques (SCP or interscalene block) may depend on the site of clavicle injury or variations in clavicular innervation. Larger prospective studies are required to further clarify the distribution of sensory blockade and the efficacy and safety of the CPB.
